# Perioperative rehabilitation in operation for lung cancer (PROLUCA) – rationale and design

**DOI:** 10.1186/1471-2407-14-404

**Published:** 2014-06-04

**Authors:** Maja S Sommer, Karen Trier, Jette Vibe-Petersen, Malene Missel, Merete Christensen, Klaus R Larsen, Seppo W Langer, Carsten Hendriksen, Paul Clementsen, Jesper H Pedersen, Henning Langberg

**Affiliations:** 1 Copenhagen Centre for Cancer and Health, Municipality of Copenhagen, Nørre Allé 45, DK-2200 Copenhagen, Denmark; 2 Department of Cardiothoracic Surgery RT, Copenhagen University Hospital (Rigshospitalet), Blegdamsvej 9, DK- 2100, Copenhagen, Denmark; 3 Pulmonary Department L, Bispebjerg Hospital, University of Copenhagen, Bispebjerg Bakke 23, DK-2400 Copenhagen, Denmark; 4 Department of Oncology, Copenhagen University Hospital (Rigshospitalet), Blegdamsvej 9, DK - 2100 Copenhagen, Denmark; 5 Department of Public Health, Section of Social Medicine, Copenhagen University, Øster Farimagsgade 5, postbox 2099, DK-1014 Copenhagen, Denmark; 6 Department of Pulmonary Medicine, Copenhagen University Hospital Gentofte, Niels Andersens Vej 65, DK-2900 Hellerup, Denmark; 7 CopenRehab, Section of Social Medicine, Department of Public Health and Centre for Healthy Ageing, Faculty of Heath Sciences, University of Copenhagen, Copenhagen, Denmark

**Keywords:** Cancer, Rehabilitation, Exercise, Lung cancer, NSCLC

## Abstract

**Background:**

The purpose of the PROLUCA study is to investigate the efficacy of preoperative and early postoperative rehabilitation in a non-hospital setting in patients with operable lung cancer with special focus on exercise.

**Methods:**

Using a 2x2 factorial design with continuous effect endpoint (Maximal Oxygen Uptake (VO_2_peak)), 380 patients with non-small cell lung cancer (NSCLC) stage I-IIIa referred for surgical resection will be randomly assigned to one of four groups: (1) preoperative and early postoperative rehabilitation (starting two weeks after surgery); (2) preoperative and late postoperative rehabilitation (starting six weeks after surgery); (3) early postoperative rehabilitation alone; (4) today’s standard care which is postoperative rehabilitation initiated six weeks after surgery. The preoperative rehabilitation program consists of an individually designed, 30-minute home-based exercise program performed daily. The postoperative rehabilitation program consists of a supervised group exercise program comprising cardiovascular and resistance training two-hour weekly for 12 weeks combined with individual counseling. The primary study endpoint is VO_2_peak and secondary endpoints include: Six-minute walk distance (6MWD), one-repetition-maximum (1RM), pulmonary function, patient-reported outcomes (PROs) on health-related quality of life (HRQoL), symptoms and side effects of the cancer disease and the treatment of the disease, anxiety, depression, wellbeing, lifestyle, hospitalization time, sick leave, work status, postoperative complications (up to 30 days after surgery) and survival. Endpoints will be assessed at baseline, the day before surgery, pre-intervention, post-intervention, six months after surgery and one year after surgery.

**Discussion:**

The results of the PROLUCA study may potentially contribute to the identification of the optimal perioperative rehabilitation for operable lung cancer patients focusing on exercise initiated immediately after diagnosis and rehabilitation shortly after surgery.

**Trial Registration:**

NCT01893580

## Background

Lung cancer is one of the most frequently occurring cancer diagnoses with the highest mortality rate [1]. Lung cancer is divided into Small-Cell Lung Carcinoma (SCLC) and Non-Small Cell Lung Carcinoma (NSCLC). Surgery is at present the primary treatment for NSCLC. According to the Danish Register of Lung Cancer 2011, the 2-year survival was 62% and the 5-year survival 42% following radical surgery for lung cancer [2]. Modern surgical treatment includes both minimal invasive surgery, e.g. video-assisted thoracoscopic surgery (VATS), and open surgery such as thoracotomy. An increasing proportion of lung cancer patients are operated by VATS technique in both Europe and the US. At Copenhagen University Hospital (Rigshospitalet), more than 60% of all lung cancers patients are operated by VATS [2].

Improved surgical techniques combined with effective adjuvant chemotherapy have led to a significant survival benefit in individuals with NSCLC [3,4]. Postoperative complications are experienced by 25% of the patients with NSCLC [2], and the risk of developing postoperative complications during the first two weeks after surgery has been reported to be dependent on different factors, e.g. preoperative cardiorespiratory capacity, measured as VO_2_peak [1,5]. The physiological consequences of ageing and inactivity combined with the cancer disease and the treatment of cancer result in a marked reduction in VO_2_peak and functional capacity [6-8]. Other factors such as smoking [9], alcohol consumption [10], nutritional status [11] and comorbidity [12] are predictors of postoperative complications. The treatment of NSCLC and other types of cancer is complex and potentially lethal. Accordingly, side effects are now recognized as a subject of major clinical importance [13]. The side effects may comprise physical and psychological as well as social distress with symptoms such as reduced cardiorespiratory capacity, paresthesia, post-thoracotomy pain syndrome, fatigue, anxiety, and depression [14-16]. The late side effects are long-lasting or even chronic and may result in restrictions in activity of daily living and reduced quality of life [5,17-23].

A Cochrane systematic review from 2012 indicates that exercise in patients with a variety of cancer diagnoses may have beneficial effects on HRQoL [24]. This is supported by a Danish randomized controlled trial with 269 cancer patients (different diagnoses) according to which patients receiving chemotherapy tolerate intensive physical exercise and experience reduced fatigue, depression, and nausea [25]. In general, rehabilitation in cancer patients based on physical exercise perioperatively has been shown to increase HRQoL and physical activity, and at the same time reduce the side effects of the treatment [24,26-33]. There is consistent evidence from 27 observational studies that physical activity is associated with reduced all-cause, breast cancer-specific, and colon cancer-specific mortality [34].

Clinical studies of *preoperative* physical exercise in patients with operable NSCLC are sparse. However, a recent prospective feasibility study on 25 patients with NSCLC reports that the patients tolerate 30 minutes of preoperative intensive cardiovascular exercise 5 times/week. The study finds that exercise significantly improves VO_2_peak and 6MWD [35]. Two other studies indicate that rehabilitation including preoperative exercise can improve physical and psychological outcome in patients with NSCLC [36,37].

The effect of *postoperative* physical exercise in patients with lung cancer has been investigated briefly. The studies differ in type of intervention, dose and timing of intervention, and the research is primarily based on case studies and studies with few and heterogeneous participants [36]. Two non-randomized feasibility studies observed that supervised moderate to high intensity cardiovascular exercise initiated four weeks after surgery is safe and feasible for operable lung cancer patients. The intervention consisted of three weekly cycling sessions for a period of 14 weeks, and participation was associated with a significantly improved HRQoL [38,39].

In a prospective study of 45 lung cancer patients, exercise on ergometer bikes 30 minutes daily, initiated two weeks after end of cancer treatment (including both surgery and chemotherapy), was reported to result in a pronounced improvement in exercise capacity and functional status [40]. The results are confirmed by other studies [41,42]. Another randomized study of 53 lobectomized lung cancer patients showed retention of muscle strength in the intervention group in which the patients participated in mobilization and strength exercise twice daily during admission followed by a 12-week long home exercise program. HRQoL (EORTC questionnaire) and physical capacity (measured by 6MWD) were unchanged [43]. Overall, these studies indicated that postoperative exercise may have a positive effect on physical capacity and HRQoL in NSCLC. A systematic review from 2011 concluded that pre- and postoperative exercise is safe and feasible for NSCLC patients and associated with a positive effect on physical capacity and, to some extent, HRQoL [26]. However, the main part of the studies quoted in the review are small case series and the only randomized study in the review observes no difference between the intervention and the control group [26]. In summary, several studies indicate that postoperative exercise of NSCLC patients is safe and associated with improvement of fitness and self-reported outcome such as HRQoL and fatigue [27,44]. Positive effects of perioperative exercise interventions are more pronounced with moderate- to vigorous-intensity versus mild-intensity exercise programs. More research is required to fully understand the potential effect of exercise over time and to determine essential attributes of exercise (mode, intensity, frequency, duration, and timing) by cancer type and cancer treatment [24].

To our knowledge the present Perioperative Rehabilitation in Operation for LUng CAncer (PROLUCA) study is the first study to investigate the clinical effects of pre- and early postoperative rehabilitation in NSCLC patients. In PROLUCA a randomized clinical trial, the efficacy of pre- and early postoperative rehabilitation is compared with the effect of rehabilitation initiated six weeks after surgery (usual care) in a non-hospital setting.

The aim of PROLUCA is to identify the optimal timing of exercise to improve VO_2_peak in postoperative NSCLC patients. The specific aims are: (1) comparison of combined preoperative home-based exercise with postoperative exercise regarding VO_2_peak and patient-reported outcomes (PROs), (2) comparison of early postoperative exercise (initiated as early as two weeks after surgery) with usual care regarding VO_2_peak and PROs.

## Methods

### Participants and settings

The study will recruit and randomize 380 patients (95 patients/study arm) with histologically or cytologically confirmed NSCLC, stage I-IIIa (TNM classification v. 7 [45]) or strong substantiated suspicion of NSCLC, referred for surgery. All subjects are assigned for curative lung cancer surgery at Department of Cardiothoracic Surgery, Copenhagen University Hospital (Rigshospitalet). The inclusion and exclusion criteria are described in Table 1: Subject Eligibility Criteria in the PROLUCA Trial.

**Table 1:** Subject eligibility criteria in the PROLUCA trial

**Inclusion criteria**	
All subjects are assigned for curative lung cancer surgery at Department of Cardiothoracic Surgery RT at Copenhagen University Hospital (Rigshospitalet).	Screened for eligibility criteria at Bispebjerg University Hospital and Gentofte Hospital.
At least 18 years old.	
Performance status 0–2 (WHO) [46].	Capable of participating in the described tests and intervention.
Living in the City of Copenhagen or surrounding Municipalities.	Reside within driving distance of Copenhagen Centre for Cancer and Health and capable of managing transportation as necessitated by the clinic-based assessments and supervised exercise interventions.
Ability to read and understand Danish.	
Approval by primary surgeon.	To examine for any contraindication in participating in physical exercise.
**Exclusion criteria**	
Presence of metastatic disease or surgical inoperability.	
Diagnosis of Lung Cancer not verified by histological diagnosis.	
Cardiac disease [6,47].	Decompensated heart failure, severe aortic stenosis, uncontrolled arrhythmia, acute coronary syndrome.
Contraindications to maximal exercise testing as recommended by the American Thoracic Society and exercise testing guidelines for cancer patients [48].	

### Procedure

The study is conducted in accordance with the CONSORT (Consolidated Standards of Reporting Trials) statement for non-pharmacologic interventions and the Helsinki Declaration [49]. Informed consent is obtained from all participants prior to initiation of any study procedures. The study is approved by The Danish National Committee on Health Research Ethics (H-3-2012-028) and the Danish Data Protection Agency (2007-58-0015).

**Figure 1:**
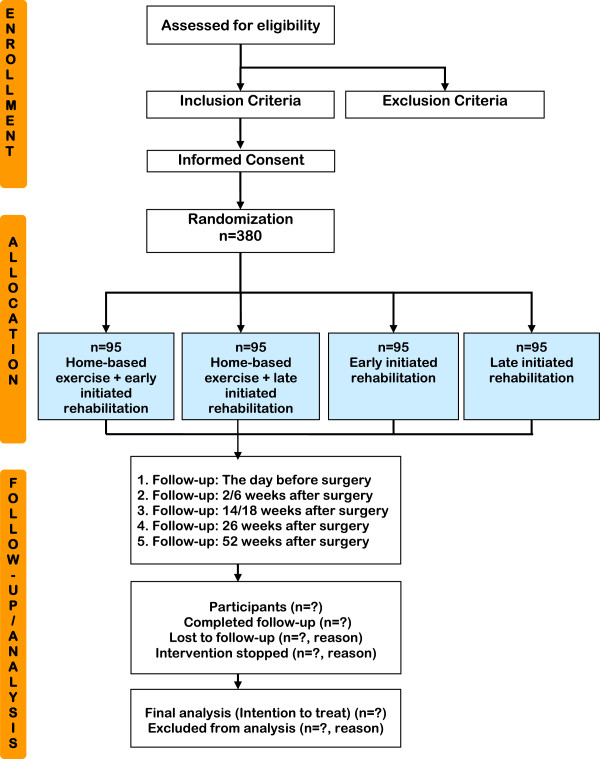
Study Flow PROLUCA.

The study flow is presented in Figure 1 Study Flow PROLUCA. Using a 4-arm, randomized design, potential subjects will be identified and screened for eligibility and informed about PROLUCA by the study research coordinators at the involved hospitals (Bispebjerg University Hospital and Gentofte Hospital). After referral to intended curative lung cancer surgery at Copenhagen University Hospital (Rigshospitalet), the subjects are contacted by telephone and provided with a review of the study. If the subjects accept to participate, the baseline assessment is performed at Copenhagen Centre for Cancer and Health. At baseline the following assessments are performed: (1) PROs described in Table 2 Data Assessment Schedule in the PROLUCA Trial, (2) anthropometric data, (3) 6MWD, (4) muscle strength (1RM in chest- and leg-press machines), (5) pulmonary function test, and (6) cardiopulmonary exercise test (VO_2_peak). All baseline assessments will be completed as close to time of diagnosis as possible and repeated the day before surgery, pre-intervention (6MWD, pulmonary function, FACT-L), post-intervention, and at follow-up six months and one year after surgery.

**Table 2:** Data assessment schedule in the PROLUCA trial

	**Baseline**^**a**^	**Flw-up**^**b**^	**Flw-up**^**c**^	**Flw-up**^**d**^	**Flw-up**^**e**^	**Flw-up**^**f**^
Anthropometric data and cancer disease	X	X		X	X	X
	**Physiological measurements**					
Cardiorespiratory capacity (VO_2_peak)	X	X		X	X	X
Six- minute walk distance (6MWD)	X	X	X	X	X	X
One-repetition-maximum (1RM)	X	X		X	X	X
Heart rate (HR), Blood pressure (BP)	X	X		X	X	X
Spirometric (FEV_1_/FEV_1_%)	X	X		X	X	X
	**Patient-reported outcome**					
Health-related quality of life (EORTC QLQ-C30, FACT-L)	X	X	FACT-L	X	X	X
Symptoms and side effects (EORTC–LC13)	X	X		X	X	X
Anxiety and depression (HADS)	X	X		X	X	X
Well-being (SF-36)	X	X		X	X	X
Distress thermometer	X	X		X	X	X
Lifestyle	X	X		X	X	X
Sickness absence and work status	X	X		X	X	X
Social support (MSPSS)	X	X		X	X	X
	**Other measurements**					
Perioperative complications	X (30 days)	X (30 days)				
Duration of hospitalization	X (30 days)	X (30 days)				
Survival Histological diagnosis and TNM staging						X

^a^Baseline (0 week).

^b^Flw-up (Follow-up): Preoperation (the day before surgery).

^c^Flw-up (Follow-up): Pre-intervention (2/6 weeks after surgery).

^d^Flw-up (Follow-up): Post-intervention (14/18 weeks after surgery).

^e^Flw-up (Follow-up): Six months after surgery.

^f^Flw-up (Follow-up): One year after surgery.

### Group allocation (Randomization)

Following the successful completion of baseline assessments, participants will be randomly allocated, on an individual basis, to one of the four exercise intervention groups:

Group 1: Preoperative home-based exercise and postoperative rehabilitation initiated as early as two weeks after surgery.

Group 2: Preoperative home-based exercise and postoperative rehabilitation initiated six weeks after surgery.

Group 3: Postoperative rehabilitation initiated as early as two weeks after surgery.

**Figure 2:**
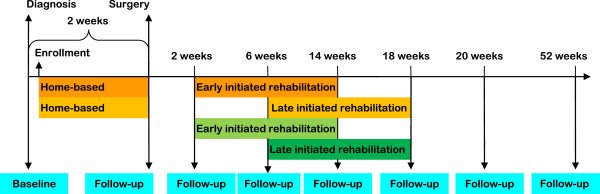
PROLUCA Study Timeline (three intervention groups and one control group).

Group 4: Postoperative rehabilitation initiated six weeks after surgery (Usual practice as control group).The random allocation sequences will be concealed from all study personnel and performed by Copenhagen Trial Unit, Centre for Clinical Intervention Research. A permuted block design with allocation weight of 1:1:1:1 will be used to generate the treatment assignments. Randomly allocated participants will remain in the same group for the entire duration of the intervention, as expressed in Figure 2 PROLUCA Study Timeline. To ensure similarity of randomized groups at baseline, patient randomization will be stratified based on type of surgery, VATS versus thoracotomy surgery.

### Blinding

It is not possible to blind the participants to their actual treatment allocation, since participants are aware whether they initiate preoperative exercise and whether their postoperative exercise starts two or six weeks after surgery. All study personnel collecting data and doing the statistical analyses of the data are, however, blinded to the patient allocation, and the patients are strictly informed not to reveal their group allocation to the test personnel.

### Exercise training protocols (General considerations)

#### ***Preoperative exercise***

The home-based exercise program is individually designed for each of the participants randomized to a preoperative intervention. The ultimate goal of the preoperative home-based exercise program is to ensure that the patients perform cardiovascular exercise of moderate-vigorous intensity (~60-80% of maximum heart rate (HRmax)) for at least 30 minutes every day until surgery. The preoperative period varies in length and the intention is not to exceed 14 days. The preoperative exercise is monitored by a heart rate sensor (Polar Team 2 System, with off-line transmitters) and an exercise diary logbook.

#### ***Postoperative rehabilitation***

The postoperative intervention consists of a supervised 12-week rehabilitation program containing 24 group-based exercise sessions, three individual counseling sessions, and three group-based lessons in health-promoting behavior. If the participants have special needs in terms of smoking cessation, nutritional counseling or patient education, this is offered too.

The postoperative physical exercise consists of an individually prepared supervised strength exercise – and a group-based cardiovascular exercise twice a week (60 minutes/session) on non-consecutive days for 12 weeks, a total of 24 sessions, containing the following elements:

Warm-up (five minutes) and cardiovascular exercise (25 minutes) on ergometer bike (BODY BIKE Classic Supreme©), individually prepared strength exercise (25 minutes) carried out using five machines (Technogym™), leg press, chest press, leg extension, pull to chest, pull-down (upper body). The practical aim of strength exercise is to complete three series of 5–12 sets. Trained physiotherapists and cancer nurse specialists supervise the training program following the recommended principles [50]. All exercise sessions will include supervised breathing exercises combined with stretching and relaxation techniques (five minutes). All the cardiorespiratory exercise is designed such that participants begin exercising at a low intensity (~50%-60% of individually determined HRmax) which is subsequently increased to more moderate to vigorous intensity (~70%-80% of individually determined HRmax). The ultimate goal for the postoperative exercise is two group-based exercise sessions per week, with a cardiorespiratory intensity for the first four weeks at ~50-60% of individual HRmax. The next eight weeks the intensity increases to moderate-high intensity at ~70-90% of individually determined HRmax. The heart rate will be monitored continuously throughout the cardiorespiratory exercise using heart rate monitors and software (Polar Team^2^ Pro©). All interventions will be individually tailored to each participant and following the principles of aerobic or resistance training prescription guidelines for adults as recommended by the American College of Sports Medicine (ACSM) [50]. The ultimate goal for the strength exercise program is to exercise with an intensity of ~60-80% of 1 RM two times a week for 12 weeks. To ensure progression every second week, the load is progressively increased and the number of repetitions are reduced starting out at 12 repetitions in three sets progressing to 10 repetitions in three sets to a final of eight repetitions in three sets. The progression is documented in a study exercise log file for registration of the intensity of all sessions along with data on blood pressure prior to exercise.

#### ***Adherence Considerations***

To maximize adherence, several strategies will be employed including telephone-based follow-ups. The patients are provided free parking in front of the center and transport expenses are covered. The high degree of scheduling flexibility allows participants to perform test at a convenient time and work around other competing demands such as medical appointments, work, and family commitments.

### Study endpoints and assessments

#### ***Primary endpoint***

VO_2_peak is evaluated by an incremental test using an electromagnetically braked cycle ergometer (Lode Corival Ergometer©). Inspired and expired gases are analyzed breath-by-breath by a metabolic cart (JAEGER MasterScreen CPX©). Subjects begin pedaling at seven watts and resistance increases after a predefined 10 watts ramp protocol until exhaustion or a symptom-limited VO_2_peak is achieved (pain, dizziness, anxiety etc.). This regimen has previously been demonstrated to be appropriate for measuring VO_2_peak in prior studies in patients with ankylosing spondylitis [51]. Other similar VO_2peak_ protocols are found appropriate for measuring VO_2_peak in NSCLC [38,39,52].

#### ***Secondary endpoints***

##### 

**Patient-reported outcomes (PROs)** PROs will include HRQoL, symptoms and side effects, anxiety and depression, well-being, distress, lifestyle, sickness absence, work status, and social support. HRQoL is assessed using the integrated system of the European Organization for Research and Treatment in Cancer (EORTC) for assessing the HRQoL of cancer patients participating in international clinical trials and devised through collaborative research. The EORTC QLQ-C30 assesses patient symptoms and HRQoL in lung cancer patients [53]. Symptoms and side effects will be assessed using the EORTC–LC13, which is an additional page to the EORTC-QLQ specifically designed to cover a wide range of lung cancer patients varying in disease stage and treatment modality [54]. EORTC measures single items and the scales range in score from 0 to 100. A high scale score represents a higher response level. A high score for a functional scale represents a high/healthy level of functioning and a high score for the global health status/quality of life represents a high HRQoL. However, a high score for a symptom scale/item represents a high level of symptomatology or problems [53,54].

HRQoL will also be assessed using the Functional Assessment of Cancer Therapy - Lung (FACT-L) scale that contains four subscales for physical (7-items), functional (7-items), emotional (6-items), social/family well-being (7-items) plus a lung cancer specific subscale (15-items) which will be summed to obtain the FACT-L score (all 42 items) [55].

General well-being is assessed using the 36-Item Short Form Health Survey (SF-36), standard recall (four weeks). The SF-36 includes eight scales measuring general health with two summary scales; physical and mental component scales [56,57]. To assess psychological well-being, the Hospital Anxiety and Depression Scale (HADS) with 14 items will be administered, designed to measure general anxiety and depression for use in investigations of patients with physical illness [58]. The distress thermometer is a validated measure of distress and consists of a single item, with responses ranging from 0 to 10 [59].

The Multidimensional Scale of Perceived Social support (MSPSS) is a 12-item scale assessing social support. Each item is answered on a seven-point Likert scale, from one: Very strongly disagree, to seven: Very strongly agree. The scale yields three subscale scores, for Family, Friends, and Significant Others, and a Total score, which is confirmed in a confirmatory factor analysis [60]. In other different cancer studies, all the above-mentioned validated instruments were found appropriate and easy to administer [35,52,59,61].

#### ***Physiological measurements***

Physiological measurements will include: (1) functional capacity, (2) pulmonary function, (3) cardiovascular O_2_ delivery, and (4) muscle strength. (1) functional capacity will be measured by a six-minute walking distance (6MWD) test carried out over a pre-measured distance of 22 m and in accordance with the American Thoracic Society (ATS) statement [62]. The 6MWD test has demonstrated good reliability and validity in patients with chronic obstructive lung disease [63], a patient group with similar symptomatology and pathophysiology. (2) Pulmonary Function will be determined by assessing the Forced Expiratory Volume in one second (FEV_1_), and the FEV_1_% which is the ratio of FEV_1_ to the Forced Vital Capacity (FVC) using a Triple V digital volume sensor© connected to JAEGER MasterScreen CPX©. All pulmonary function tests will be performed in a standing position and according to the ATS guidelines [48]. (3) Arterial O_2_ Saturation will be assessed at rest and continuously during exercise using pulse oximetry (Nellcor, OxiMax N-65©), which provides the most accurate non-invasive assessment of blood arterial O_2_ saturation levels. Muscle strength is measured by 1RM [64] using machines (Technogym™) that includes leg press (lower extremity) and chest press (pectoral muscles).

#### ***Disease-related outcomes***

In all patients lung cancer subtype, stage and extent of surgery will be related to the effect of exercise and rehabilitation interventions.

#### ***Tracking and monitoring of adverse events***

Tracking and monitoring of adverse events are assessed as follows: (1) before every intervention- and test-session, all patients will receive face-to-face supervision by a specialized trained cancer nurse discussing any potential negative side effects of the intervention assignment. All injuries and adverse events (e.g., knee pain, back pain) will be recorded as unintended events. In addition, heart rate and blood pressure are recorded prior to every intervention session and repeated if any adverse events should occur during exercise.

### Statistical considerations

#### ***Sample size calculation***

This randomized phase II trial will accrue 380 subjects with operable NSCLC over an accrual period of ~2 years. The present design consists of four intervention groups of equal size, and it is assumed that no interaction occurs between the groups. With the smallest clinical relevant difference set at 2 mLO_2_^.^ kg^-1 .^ min^-1^,95 participants are required in each group (power: 80%), giving a total inclusion of 380 patients.

For each of the primary and secondary endpoints, three separate t-tests will be used to compare each experimental arm to the control arm in mean change across time of the endpoint. For each endpoint, the overall alpha level will be controlled at a two-sided 0.05 by using Holm's procedure [65]. That is, Holm's procedure first ranks the three p-values from lowest to highest. The first (lowest) p-value has to be less than 0.05/3 (0.0167) to be significant and permit continuation to the other t-tests. The Holm's procedure continues sequentially in this fashion using alpha levels of 0.05/2 (0.025) and 0.05/1 (0.05) for the remaining two t-tests, respectively. Power for this study is defined as the probability that at least one of the three t-tests of the arm effect on VO_2_peak is significant; in other words, power is the probability that the first of the 3 ordered t-tests are significant. We assume that change in VO_2_peak will have a standard deviation of 4.0 mL ^.^ kg^-1 .^ min^-1^ as observed in previous research [27,36]. Statistical power depends upon the configuration of mean change in VO_2_peak across the 4 arms. Thus, for example, 80% power is obtained when the mean change in VO_2_peak across Arms 1, 2, 3, and 4 is 0.60, 0.60, 2.10, and 0.0 (mL ^.^ kg^-1 .^ min^-1^), respectively.

#### ***Analytic plan***

The intention-to-treat analysis includes all randomized participants in their randomly assigned allocations. The intervention group assignment will not be altered based on the participant's adherence to the randomly allocated study arm. Patients who are lost-to-follow-up are included in the analysis (intention to treat). For the primary analysis, a multiple regression model will be used to assess a change in VO_2_peak on study group, the baseline value of the endpoint, and other pertinent baseline variables that may influence change in the study endpoints (e.g., co-morbid conditions/medications, self-reported exercise history, age). Data from PROs will be presented as mean, standard deviation (SD), median and inter-quartile range (IQR) and all change scores (value at follow-up minus value at baseline) will be presented with a 95% confidence interval.

## Discussion

The aim of PROLUCA is to contribute with important knowledge about the efficacy of pre- and early postoperative rehabilitation in patients with NSCLC in a non-hospital setting.

The decision to target newly diagnosed patients with NSCLC was primarily based on the fact that these patients are not often examined in relation to the effect of rehabilitation, although they generally have a good performance status and prognosis after surgery and adjuvant chemotherapy. In consequence, the issue of NSCLC survivorship is becoming an increasingly important aspect of the multidisciplinary care of this patient group and the demands for knowledge correspondingly important.

The need for rehabilitation becomes obvious by the fact that NSCLC patients are subject to a marked decrease in cardiorespiratory capacity due to a combination of age and comorbidity and reinforced by the use of adjuvant cancer treatment [6]. It is well known that good preoperative cardiorespiratory capacity leads to better postoperative conditions resulting in less postoperative complications in patients with NSCLC [1,5]. Further studies are also warranted on other physical effects of exercise and how to commit this group of cancer patients to a more active lifestyle.

Studies focusing on the effects of exercise interventions pre- and postoperatively are required to fully understand the potential effect of exercise over time. The optimal characteristics of exercise (mode, intensity, frequency, duration, and timing) have yet to be determined [24].

No published research in cancer rehabilitation has investigated the best timing of rehabilitation in patients with NSCLC in a randomized clinical trial. Qualitative studies have pointed out that cancer patients may experience transition points during time of illness to which they are particularly vulnerable: (1) diagnosis, (2) operation and hospitalization, (3) transition from hospital to daily life, and (4) return to daily life [66-72]. The timing of rehabilitation has also been indicated to of importance when it comes to motivation toward a healthier life style in patients with a variety of cancer diagnoses [73]. The ‘teachable moment’ is a term used in e.g. research in breast cancer patients describing the transition that takes place when the patients are diagnosed. This transition can modify barriers and motivate the patient; thus timing of rehabilitation is of great importance for the outcome [74].

The PROLUCA study aims at revealing the impact of timing of rehabilitation on VO_2_peak and health-promoting behavior in patients with NSCLC. The effect six months and one year after surgery is measured. VO_2_peak is chosen as the primary endpoint as this test provides the gold standard (direct) assessment of cardiorespiratory capacity [13]. In a hospital setting it would have been interesting to test cardiorespiratory capacity as early as two weeks postoperatively, but as the intervention in PROLUCA is carried out in a non-hospital setting, this is not possible due to safety reasons. According to the Danish Health Act from 2007 the responsibility for rehabilitation of all patients with a decrease in functional capacity lies with the municipalities unless medical assistance is needed. The same is true of patient-targeted prevention.

The patients perform a VO_2_peak test preoperatively and again after the intervention. The first test acts as a surrogate parameter for the starting point, and PROLUCA is therefore not capable of clarifying what happens to VO_2_peak shortly after surgery. Research indicates that VO_2_peak spontaneously recovers to a limited degree at approximately 3 months after surgery and stabilizes at approximately 6 months after pulmonary resection. Another study finds a 13% decrease in VO_2_peak ~6 months after surgery [75]. As this study compares the preoperative VO_2_peak with postoperative VO_2_peak value 6 months after surgery, the best estimate possible is chosen.

To obtain a patient population as close to normal daily practice as possible where patients are suffering from a variety of comorbidity, PROLUCA limits the amount of exclusion criteria. This makes PROLUCA unique compared to other studies whose selection of patients is distinct. Therefore the results of PROLUCA may contribute importantly to daily clinical practice.

With the increasing interest in the field of exercise-oncology research, more studies are now focusing on the application of exercise as a concomitant intervention alongside anti-cancer therapies.

### Summary

Even though rehabilitation, with focus on exercise, is widely recommended to cancer patients, information concerning timing and dose of exercise rehabilitation is lacking when it comes to patients operated for NSCLC. To our knowledge no previous studies have been published in which postoperative rehabilitation is initiated as early as two weeks after surgery for NSCLC. Furthermore, there is a distinct need for trials including NSCLC patients, since this group of patients is especially vulnerable due to a high burden of comorbidity, risk of relapse of the cancer disease and consequences of both surgical and oncological treatment. In addition, the patient population included in PROLUCA is as close to those seen in normal daily practice as possible. This makes PROLUCA unique compared to other studies whose selection of patients is distinct, and the results of the PROLUCA study may contribute importantly to daily clinical practice.

## Abbreviations

VO_2_peak: Maximal oxygen uptake; NSCLC: Non-small cell lung carcinoma; HRmax: Heart rate max; 6 MWD: Six-minute walk distance; 1 RM: One-repetition-maximum; PROs: Patient-reported outcomes; HRQoL: Health related quality of life; SCLC: Small-cell lung carcinoma; VATS: Video-assisted thoracoscopic surgery; TNM: Tumor node metastasis; ACSM: American College of Sports Medicine; EORTC: The European Organization for Research and Treatment in Cancer; FACT-L: Functional assessment of cancer therapy – lung; SF-36: 36-item short form health survey; HADS: Hospital anxiety and depression scale; ATS: American Thoracic Society; FEV1: Forced expiratory volume in one second; FVC: Forced vital capacity; FEV_1_%: Which is the ratio of FEV_1_ to the FVC.

## Competing interests

The authors declare that they have no competing interests.

## Authors’ contributions

MSS: conception and design, drafting of manuscript and final approval for publication. KT: conception and design, drafting of manuscript and final approval for publication. JVP: conception and design, drafting of manuscript and final approval for publication. MM: conception and design and final approval for publication. MC: conception and design and final approval for publication. KRL: conception and design and final approval for publication. SWL: conception and design and final approval for publication. CH: conception and design and final approval for publication. PC: conception and design and final approval for publication. JHP: conception and design and final approval for publication. HL: conception and design and final approval for publication. All authors read and approved the final manuscript.

## Pre-publication history

The pre-publication history for this paper can be accessed here:

http://www.biomedcentral.com/1471-2407/14/404/prepub
